# *TM8* represses developmental timing in *Nicotiana benthamiana* and has functionally diversified in angiosperms

**DOI:** 10.1186/s12870-018-1349-7

**Published:** 2018-06-22

**Authors:** Heleen Coenen, Tom Viaene, Michiel Vandenbussche, Koen Geuten

**Affiliations:** 1 Department of Biology, KU Leuven, Kasteelpark Arenberg 31, B-3001 Leuven, Belgium; 2 Laboratoire Reproduction et Développement des Plantes, University Lyon, ENS de Lyon, UCB Lyon 1, CNRS, INRA, F-69342 Lyon, France

**Keywords:** *TM8*, *SVP*, VIGS, *Nicotiana benthamiana*, Repressor of miR172, RNAi, Overexpression, *Solanum lycopersium*, *Petunia hybrida*

## Abstract

**Background:**

MADS-box genes are key regulators of plant reproductive development and members of most lineages of this gene family have been extensively studied. However, the function and diversification of the ancient *TM8* lineage remains elusive to date. The available data suggest a possible function in flower development in tomato and fast evolution through numerous gene loss events in flowering plants.

**Results:**

We show the broad conservation of *TM8* within angiosperms and find that in contrast to other MADS-box gene lineages, no gene duplicates have been retained after major whole genome duplication events. Through knock-down of *NbTM8* by virus induced gene silencing in *Nicotiana benthamiana*, we show that *NbTM8* represses *miR172* together with another MADS-box gene, *SHORT VEGETATIVE PHASE* (*NbSVP*). In the closely related species *Petunia hybrida*, *PhTM8* is not expressed under the conditions we investigated and consistent with this, a knock-out mutant did not show a phenotype. Finally, we generated transgenic tomato plants in which *TM8* was silenced or ectopically expressed, but these plants did not display a clear phenotype. Therefore, no clear function could be confirmed for *Solanum lycopersium*.

**Conclusions:**

While the presence of *TM8* is generally conserved, it remains difficult to propose a general function in angiosperms. Based on all the available data to date, supplemented with our own results, *TM8* function seems to have diversified quickly throughout angiosperms and acts as repressor of *miR172* in *Nicotiana benthamiana*, together with *NbSVP*.

**Electronic supplementary material:**

The online version of this article (10.1186/s12870-018-1349-7) contains supplementary material, which is available to authorized users.

## Background

MIKC^C^-type MADS-box genes are plant transcription factors involved in diverse developmental processes [1–3]. In gymnosperms, 12 clades can be distinguished while 17 clades are present in angiosperms as a result of duplication events [4]. MICK^C^-type MADS-box genes are generally known for their roles in the flowering transition, floral meristem and floral organ identity, fruit and seed development. Their key role in plant reproductive processes, often of interest for crop improvement, has led to the functional characterization of most members in at least one species. The function of a few MIKC^C^-type lineages, however, remains elusive, and the *Tomato MADS 8*-clade *(TM8)* is one of these [5].

Because of its absence in classical model species such as *Arabidopsis*, maize and rice, *TM8* was originally not functionally characterized in any species. A recent phylogenomic study evaluating the evolutionary conservation of MICK-type MADS box genes in angiosperms, describes five independent losses of the *TM8*-clade throughout the angiosperms, making it the most often lost MADS-box lineage, next to *FLOWERING LOCUS C* (*FLC*) [6]. On the other hand, as a result of the increasing number of available genomes and sequences, it became clear that *TM8* homologs appear to be present throughout the spermatophytes.

*Tomato MADS 8* (*TM8*) was the first member of the clade to be identified when it was isolated from *Solanum lycopersicum.* It was classified as an ‘early’ flowering gene, expressed in floral meristems and to a lesser extent in the three inner floral whorls [7]. Its first characterization was also in tomato. Overexpression of full length *TM8*-antisense RNA resulted in defects of the female floral organs, male and female sterility and parthenocarpy in three out of 12 transgenic lines [8]. More recently, in an effort to thoroughly characterize *TM8*, Daminato et al. produced transgenic tomatoes overexpressing full-length *TM8* and plants expressing *TM8* fused to the SRDX transcriptional repressor domain [9]. They observed malformations of stamens in three out of 22 independent *TM8* overexpression lines, and consistent with this, expression of B-genes was altered in these flowers. The 15 *35S:TM8:SRDX* lines displayed more pronounced phenotypes like epinastic darker green leaves and oblong ovaries and fruits, which were seedless. Flower and fruit peduncles appeared longer and the abscission zone was abnormal. *JOINTLESS*, an *SVP* homolog, was significantly down regulated in these plants. The authors concluded that *TM8* plays some role in the development of tomato flowers by possible interactions with *MACROCALYX*.

Aside from this study, hardly any functional data on *TM8* have been presented thus far. Few studies, mostly pursuing a genome wide analysis of MADS-box genes in a certain species, report the presence of *TM8* in a diversity of genomes and a function has sometimes been proposed based on gene expression data. In *Cucumis sativus*, *ERAF17*, the cucumber *TM8* ortholog, is involved in the development of female flowers after induction by ethylene [10]. In *Antirrhinum majus,* the *TM8* ortholog *DEFH7,* is only expressed in young bracts (unpublished observations, [11]). Hileman et al. detected *TM8* in nearly all tomato tissues, albeit to a lesser extent in roots, seedlings, carpels and green fruit [12]. In *Vitis vinifera*, *VvTM8* was detected in latent buds during the flowering transition, in later stages of flower development and in tendrils [13]. However, in a more recent transcriptional analysis of tendril and inflorescence development, *VvTM8* was not mentioned [14]. In a molecular study of fleshy fruit-like structures in gymnosperms, *TM8* was unexpectedly identified [15], again displaying a broad expression pattern in both vegetative and reproductive structures. *TbTM8* was most highly expressed during arilus development in *Taxus baccata*. In *Ginkgo biloba*, three *TM8*-like genes are expressed in leaves, but are also present in male and female cones and during seed and fleshy sarcotesta development. Based on these observations, *TM8* was assigned a role in fleshy fruit-like development in gymnosperms. In another genome-wide identification of MADS-box genes in *Prunus mume*, *PmMADS26* was identified as the sole *TM8* ortholog expressed only in the pistil and the fruit, its expression gradually increasing during ripening [16]. In the closely related *Prunus persica* on the other hand, the *TM8* ortholog, identified as *PpeMADS35*, was only very lowly expressed in roots, leaves, cotyledons, embryo’s and fruits [17]. In a study in pear, *PpTM8–1* showed high expression in the bud, branch, leaf and root but not in the flower, while *PpTM8–2* was highly expressed in all tissues [18]. A similar pattern was found in *Malus domesticus*, where one *TM8* homolog, *MdMADS045* showed expression in fruits and strong expression during flower development, while the other, *MdMADS111*, was rather lowly expressed in most tissues except in the developing fruits [19].

Though no clear general or conserved role for *TM8* can be concluded from these diverse expression data, most studies share a broad *TM8* expression pattern, both in vegetative and reproductive tissues and most often during floral meristem and fruit development. Therefore, *TM8* is most commonly assigned a function in flower and fruit development [9, 13, 15]. However, clear knock-down or knock-out mutants are not available and seem necessary to provide more insight into its true role. In this study, we evaluate the function of *TM8* function within Solanaceae by means of a *Petunia hybrida* (*Petunia*) *PhTM8* knock-out mutant, and transgenically altered *TM8* expression in *Nicotiana benthamiana* (*Nicotiana*) and *Solanum lycopersicum* (tomato). The diverse results obtained in this work combined with the miscellaneous data from previous studies listed above, support the idea of rapid functional evolution of the *TM8* gene.

## Results

### *TM8* was not retained after major genome duplication events

Recent studies showed that *TM8* is highly conserved in gymnosperms, and that this clade even expanded in gymnosperms, becoming one of the largest MADS-box clades in fruitless seed plants [4]. In angiosperms on the other hand, it is designated to be the most often lost MADS-box subclade together with *FLC* [6]. To gain further insight in its evolutionary history we reconstructed its phylogeny in angiosperms, thereby not only focussing on where it was lost, but also trying to describe its widespread conservation. To do this, we used sequences from all available genomes to date in Phytozome 12 and the Sol Genomics Network (Additional file 1), supplemented with sequences from EST and transcriptome databases on NCBI and the oneKP platform [20, 21]. Special efforts were made to identify orthologs in species belonging to orders lacking fully available genomes.

**Fig. 1:**
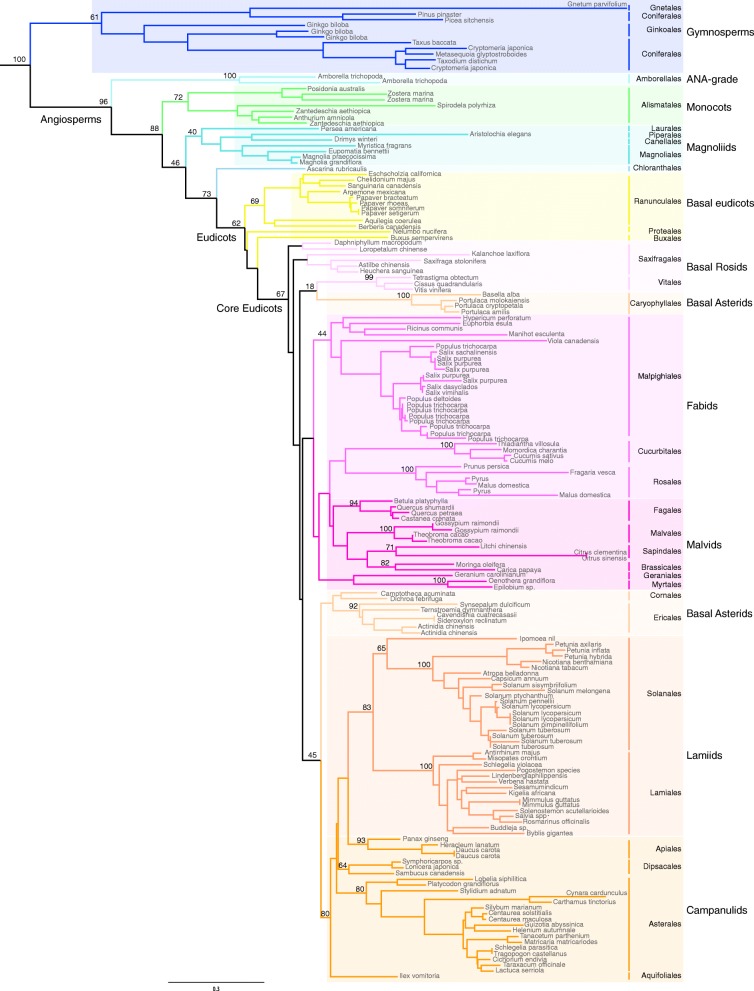
Maximum Likelihood phylogeny of the *TM8*-family in angiosperms with bootstrap support. BS support values are shown at major nodes when >50. *SOC1* genes were used as outgroup (not shown here). In angiosperm orders lacking here, no *TM8* orthologs could be identified from the databanks listed in the Methods. Complete phylogeny including all accession numbers is provided as Additional file 2

The resulting phylogeny clearly demonstrates that *TM8* is indeed present in many orders throughout the angiosperms and that the gene phylogeny follows the angiosperm phylogeny (Fig. 1, Additional file 2). This implies that no major duplications occurred within the *TM8*-lineage, which is in contrast to most other MADS-box genes which, like other transcription factors, are generally conserved after whole genome duplications [22, 23]. On the other hand, the number of cases in which *TM8* was lost or could not be identified are strikingly numerous and well spread throughout angiosperm evolution. No *TM8* orthologs could be detected in species belonging to Nymphaeales, to all monocots minus the Alismatales, to Trochodendrales, to Gunnerales, Dilleniales, Santanales, Fabales, Gentianales or Boraginales in addition to other smaller orders and many important families like the Brassicaceae. Only one or a few orthologs were identified in the Chloranthales, Proteales, Buxales, Geraniales and Cornales. While we cannot draw final conclusions about its loss in a species when no fully sequenced genome is available, to date many species are broadly sampled and have extensive transcriptome data available. The lack of *TM8* from these databases does suggest its absence in these taxa.

### *NbTM8* and *NbSVP* repress *miR172* in *Nicotiana benthamiana*

**Fig. 2:**
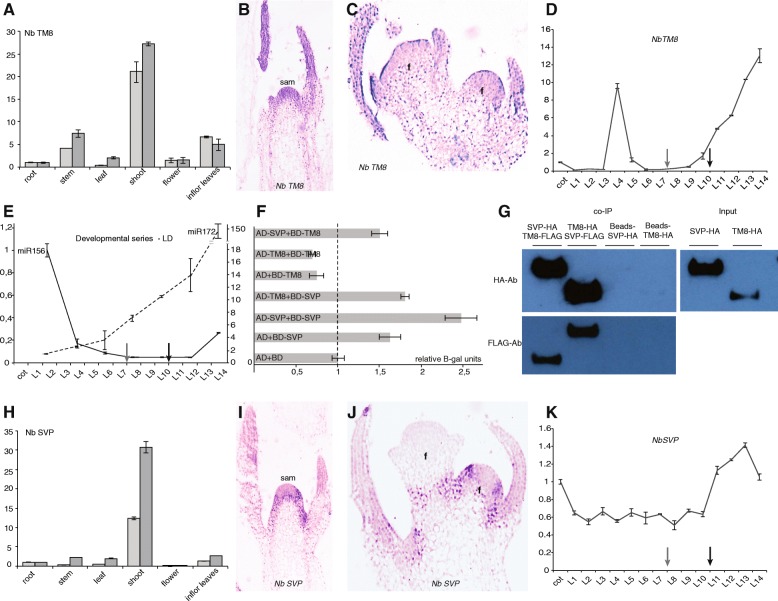
Gene expression of *NbTM8* and *NbSVP* in *Nicotiana benthamiana*. **a** Relative expression of *NbTM8* in vegetative and reproductive structures of two biological replicates. **b**–**c** In situ hybridisation of *NbTM8* in developing shoots and flowers. **d** Developmental series from cotyledons (cot) to 14 leaves (L14) of *NbTM8*. Gray arrow marks the floral transition, the black arrow marks the floral induction. **e** Relative expression of *miR156* (left) and *miR172* (right) during *Nicotiana* development. **f** Yeast two-hybrid of *NbTM8* and *NbSVP*. **g** Co-IP results for *NbTM8* and *NbSVP*. Proteins were pulled down with FLAG coupled agarose beads and visualised by HA (up) and FLAG (down) antibodies through Western Blot. **h** Relative expression of *NbSVP* in vegetative and reproductive structures. **i**–**j** In situ hybridisation of *NbSVP* in developing shoots and flowers. **k** Developmental series of *NbSVP*. sam: shoot apical meristem; f: flower meristem; LD: long day. Error bars are standard deviations

We decided to use *Nicotiana benthamiana* for the initial characterization of *TM8*, as it has been proven to be an excellent model species for virus induced gene silencing (VIGS), a rapid and easy method to obtain loss of function phenotypes [24]. To first evaluate when and where *TM8* functions in *Nicotiana*, we used qPCR and in situ hybridization (Fig. 2). The qPCR results show that *NbTM8* is strongly expressed in the stem and to a lesser extent in seedlings, leaves and floral organs (Fig. 2a). The in situ hybridizations reveal *NbTM8* expression in both developing shoots and flowers (Fig. 2b, c; Additional file 3). Expression in a developmental series of plants, each with one more leaf, shows a strong peak early in vegetative development around the initiation of the fourth leaf. Its expression drops after this peak and increases again only around the ninth or tenth leaf and continues to increase until the first flower appears (14th leaf) (Fig. 2d). We hypothesized that the first peak might be around the juvenile-adult transition, therefore we also quantified *miR156* and *miR172* levels by stem-loop qPCR (Fig. 2e). In agreement with this hypothesis we found that the first peak in *NbTM8* expression coincides almost perfectly with the moment that *miR156* drops below the increasing *miR172* level. In *Arabidopsis*, this juvenile-adult transition is characterized by the production of trichomes on the abaxial sides of leaves [25], so we studied the juvenile-adult transition in *Nicotiana* to identify similar markers for this phase change. We observed that both large trichomes and a pointed leaf tip emerge around the third and fourth leaf, confirming that the phase transition indeed takes place at this point (Additional file 4A–C). The second peak in *NbTM8* expression coincides with the increased expression of a homolog of *SUPPRESSOR OF CONSTANS 1* in *Nicotiana* (*NbSOC1*) as shown in the graph by a grey arrow (Fig. 2d, e and Additional file 4D). *SOC1* is a marker of floral induction in *Arabidopsis* and is strongly expressed during the floral transition [26]. These observations suggest that *NbTM8* acts during both phase transitions in *Nicotiana*, possibly by regulating *miR156* and *miR172*.

As MADS-box genes often function in heteromeric complexes, we considered whether a protein interaction partner might be involved in this regulation. A good candidate would be *SHORT VEGETATIVE PHASE* (*SVP*), another MADS-box gene involved in the floral transition by down regulation of *miR172* in *Arabidopsis* [27]. We first tested the ability of *NbSVP* and *NbTM8* to interact using yeast-two-hybrid. While we found a positive interaction in one direction (AD-SVP + BD-TM8), the result in the other direction (AD-TM8 + BD-SVP) remains inconclusive due to the auto-activation of NbSVP (Fig. 2f). Therefore, we subsequently performed co-immunoprecipitation from *Arabidopsis* leaf mesophyll protoplasts and confirmed that these proteins can interact in vitro (Fig. 2g).

Therefore, we decided to also characterize *NbSVP*. qPCR reveals that *NbSVP* is more strongly expressed in seedlings and leaves and to a lesser extent in the stem when compared to *NbTM8* (Fig. 2h). It seems thus that both *NbTM8* and *NbSVP* acquire their strongest expression levels in the vegetative parts of *Nicotiana benthamiana*, and that the relative expression of *NbSVP* in flowers is also lower than for *NbTM8* compared to the expression in the vegetative parts. In situ hybridization of *NbSVP* further reveals that *NbSVP* is expressed in developing shoots, similar to *NbTM8*, but in contrast, its expression is absent in floral meristems (Fig. 2i, j; Additional file 3). This is similar to *Arabidopsis* where *SVP* expression is constant during vegetative growth but reduces in the inflorescences and further disappears during flower development [28, 29]. qPCR in a developmental series reveals that in contrast to *NbTM8*, expression of *NbSVP* is continuously stronger and shows no major peaks early in vegetative development. Similar to *NbTM8*, *NbSVP* mRNA expression increases when the flowering process starts, around the time when *Nicotiana APETALA1* (*NbAP1*) is expressed (Fig. 2k, Additional file 4D).

These data on the co-expression and interaction of *NbSVP* and *NbTM8*, suggests that *NbTM8* together with *NbSVP* may have overlapping roles in the transition of the shoot to flowering. Expression of *NbTM8* early in vegetative development further suggests that *NbTM8* might play a role in the juvenile to adult transition.

**Fig. 3:**
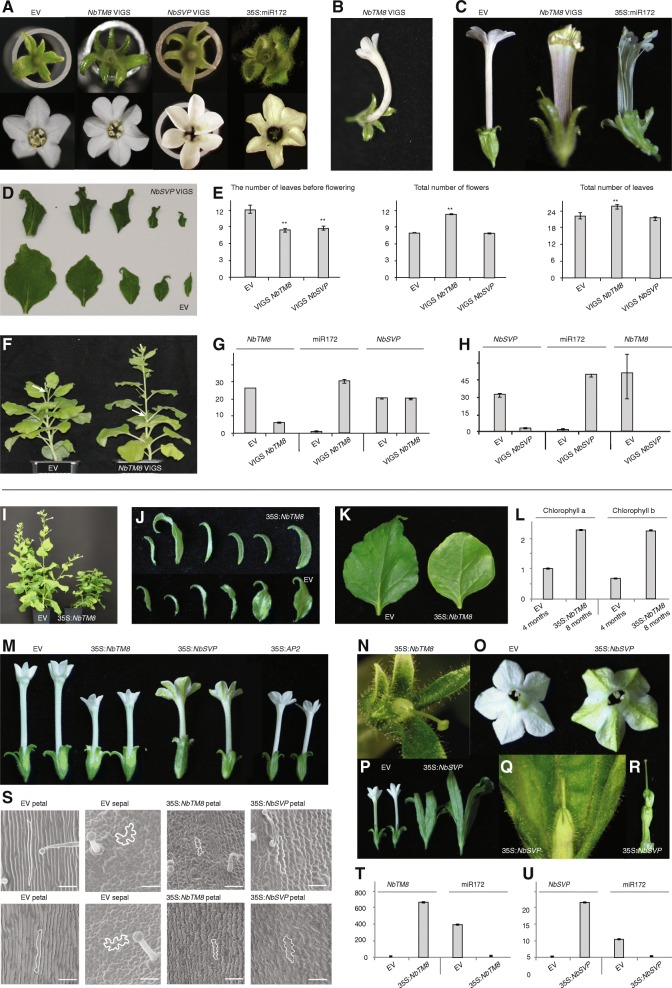
Effects of silencing and overexpression of *NbTM8* and *NbSVP*. **a** Extra floral organs in *NbTM8-* and *NbSVP*-VIGS plants, similar to the 35S:*miR172* phenotype. **b** Twisted petal tubes in some *NbTM8*-VIGS plants. **c** Split petal tubes in *NbTM8*-VIGS and 35S:*miR172* plants. **d** *NbTM8*-VIGS plants have smaller, greenisher and twisted leaves in the inflorescence. **e** Early flowering and accelerated development in *NbTM8*-VIGS and *NbSVP*-VIGS plants. Asterisks indicate significant differences at the 0.01 significance level applying a Student’s t-test. **f** *NbSVP*-VIGS plants are early flowering compared to control plants. (Arrows indicate first flowers). **g** Relative expression of *NbTM8*, *miR172* and *NbSVP* in inflorescence tissue of *NbTM8*-VIGS and control plants. **h** Relative expression of *NbSVP*, *miR172* and *NbTM8* in *NbSVP*-VIGS and control plants. **i** Strongest 35S:*NbTM8* line grows more slowly and only develops juvenile leaves. **j** Sepal and bracts of EV compared to sepal and sepaloid bracts of 35S:*NbTM8.* **k** Close-up of adult leaf compared to 35S:*NbTM8* leaf. **l** Comparison of chlorophyll levels between 4 month old EV plants and 8 month old 35S:*NbTM8* plants. **m** Reduced petal tubes in 35S:*NbTM8*, 35S:*NbSVP*, and 35S:*NbAP2* flowers. N) Strongly reduced petal tube of 35S:*NbTM8* flower. **o** Petaloid sepals in 35S:*NbSVP* lines. **p** Leaf-like sepals in 35S:*NbSVP* lines in comparison to EV. **q** Strongly reduced petal tube and enlarged sepals of 35S:*NbSVP* flower. **r** Leaf like carpels in 35S:*NbSVP* flower. **s** Scanning electron microscopy of EV sepals and petals compared to 35S:*NbTM8* and 35S:*NbSVP* petals. Scale bar represents 50 μm. **t** Relative expression levels of *NbTM8* and *miR172* in inflorescence tissue of control and 35S:*NbTM8* lines. **u** qPCR of *NbSVP* and *miR172* in inflorescence tissue of control and 35S:*NbSVP*. EV: empty vector control lines. Error bars are standard error of the mean of three technical replicates

We evaluated the effect of *NbTM8* and *NbSVP* knock down by using virus induced gene silencing. Empty vector (EV) transformed *Agrobacterium* was injected in control plants. The resulting *NbTM8*-VIGS plants and *NbSVP*-VIGS plants were remarkably similar in several ways. *NbTM8*-VIGS plants displayed a range of characteristic defects in both flowers and inflorescence leaves. The most often observed anomaly (in 15% of the VIGS flowers compared to 3% in EV, *p* = 0.018) was an increased number of floral organs in the outer three whorls of VIGS plants compared to EV plants, a characteristic also regularly displayed by *NbSVP*-VIGS plants (19% of the flowers compared to 3% in EV, *p* = 0.004) (Fig. 3a). Furthermore, in some cases, *NbTM8*-VIGS petal tubes were twisted or sometimes even open (Fig. 3b, c). Leaves in the inflorescence were smaller, twisted and darker green (Fig. 3d). Overall, *NbTM8*-VIGS plants can be described as undergoing accelerated development based on the increased rate of flower and leaf production. Both *NbTM8*-VIGS and *NbSVP*-VIGS plants were early flowering when using the number of leaves before flowering as a proxy (Fig. 3e–f). They flower early by approximately three leaves compared to control plants, suggesting that both *NbSVP* and *NbTM8* function as repressors of the floral transition in *Nicotiana* (Fig. 3e). For *NbSVP* this should not come as a surprise, as *SVP-*like genes have been shown to act as a repressor of the floral transition in *Arabidopsis thaliana* [28]. For *TM8* however, an early flowering phenotype has not yet been attributed to a member of this subfamily. qPCR confirmed much reduced *NbTM8* and *NbSVP* expression in both inflorescence leaves and flowers of *NbTM8*-VIGS and *NbSVP*-VIGS plants respectively compared to the EV control (Fig. 3g). Interestingly, the observed phenotypes strongly resemble those of *Nicotiana* plants that constitutively express *Arabidopsis miR172* (Fig. 3a–c) [30]. To verify whether increased expression of *miR172* may explain some of the phenotypes, *miR172* expression levels were quantified in *NbTM8*-VIGS and *NbSVP*-VIGS lines. The results show that *miR172* is strongly upregulated in the VIGS lines in comparison to EV control plants (Fig. 3g, h). Because *NbTM8* and *NbSVP* can interact and show similar phenotypes, they might also regulate each other. Indeed, *NbSVP* silencing results in the concomitant down regulation of *NbTM8* (Fig. 3h), while silencing of *NbTM8* does not affect the *NbSVP* level (Fig. 3g).

As silencing seemed to accelerate developmental transitions, we addressed whether constitutive expression would delay these transitions by generating transgenic *NbTM8* and *NbSVP* overexpression plants. While caution is required interpreting overexpression phenotypes due to off-target binding, we observed a number of consistent alterations. Out of all 35S:*NbTM8* lines, the strongest one showed a dramatic transformation of all leaves into juvenile leaves and inflorescence bracts remained sepal-like (Fig. 3i, j). Two lines showed a significant increase in lifespan – more than threefold – and senescence was delayed as indicated by a comparison in chlorophyll levels (Fig. 3k, l). Furthermore, these strongest lines did not produce any seed. In all transgenic lines, petal tubes were severely reduced in size and became more greenish (Fig. 3m, n), a phenotype that was previously observed in plants that constitutively express *Arabidopsis APETALA2* (*AP2*) in *Nicotiana* [30]. Transgenic plants overexpressing *NbSVP* also display similar phenotypes as 35S:*NbTM8* plants. They also resulted in reduced and greenish petal tubes (Fig. 3m, o–q). Scanning electron microscopy shows that this is a consequence of a partial petal to sepal transformation (Fig. 3s). In other 35S:*NbSVP* lines the petal tube was even more strongly reduced while sepals were enlarged (Fig. 3p, q). The sepals and carpels were leaf-like, as previously described for ectopically expressed *SVP*-like genes in other Solanaceae species (Fig. 3p–r) [31]. Both in 35S:*NbTM8* and 35S:*NbSVP* lines, *miR172* was significantly down regulated (Fig. 3t, u).

Together, *NbTM8* and *NbSVP* transcripts have overlapping expression patterns in the shoot apical meristem, in leaves, bracts and in flower meristems. Their expression patterns are uncoupled when sepal primordia emerge and *NbSVP* expression disappears. Given the above data, it is plausible that *NbTM8* and *NbSVP* act as negative regulators of *miR172* in *Nicotiana* and may control the timing of developmental transitions together by repressing *miR172* levels.

### *TM8* appears to have lost its function in *Petunia hybrida*

**Fig. 4:**
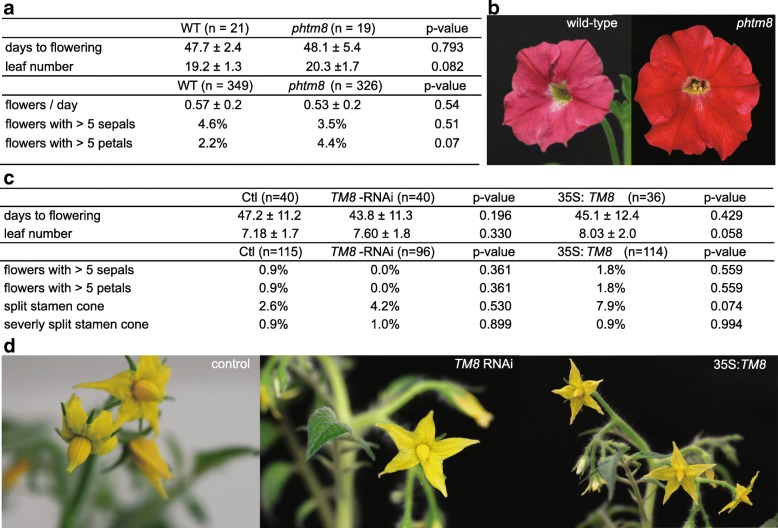
*PhTM8* mutant and *TM8* altered transgenic tomatoes do not differ from control plants. **a** Mean and standard deviation of flowering time, number of leaves before flowering or number of floral organs of the *PhTM8* knock-out mutant and wild-type. **b** No apparent difference between wild-type *Petunia* and *phtm8*. **c** Mean and standard deviation of flowering time, number of leaves before flowering, number of floral organs or anomalies in the stamen cone of transgenic tomatoes and control. **d** Control, *TM8* silenced and overexpressed tomatoes. *p*-values are determined by unpaired two-tailed Students t-test

The next step was to evaluate the conservation of this flowering repressor complex in other species. The closely related *Petunia* is an excellent model, considering the availability of a *PhTM8* knock-out mutant which contains a footprint resulting from a *dTph1* transposon insertion in the start codon, leading to a frame shift (Additional file 5). We phenotyped 19 footprint-mutant plants (*phtm8*) and 21 wild-type plants for developmental and morphological traits, but no differences in flowering time, leaf or flower morphology could be observed (Fig. 4).

To evaluate the effect of *PhTM8* overexpression, we tried to generate 35S:*PhTM8* lines but we were not able to clone *PhTM8* from *Petunia* cDNA despite of extensive cloning efforts (see methods). In a subsequent search for *PhTM8* expression data we found that *PhTM8* was also not picked up in a MADS-box gene screening of various *Petunia* cDNA libraries by Immink et al. [32].

Blasting in all available *Petunia* transcriptome and EST databases to date did not deliver any hits either, suggesting that *PhTM8* is not or only very weakly expressed in *Petunia*, at least in normal growth conditions. In the protein sequence however, no obvious deviation from other *TM8* orthologs or marks of pseudogenization can be observed (Additional file 6). The protein could therefore still be functional, but might only be expressed in conditions that haven’t been tested yet.

Taken together, *PhTM8* appears not to act as a floral repressor in *Petunia* in contrast to *NbTM8* in *Nicotiana* and its function appears to have diverged between these species belonging to the same family.

### The function in tomato also remains unclear

Since *Petunia* did not seem to express *PhTM8*, we continued our investigations in tomato, another member of the Solanceae family and the first species in which *TM8* was identified [7]. Two overexpression lines and 22 RNAi lines were obtained by *Agrobacterium*-mediated transformation. Control lines were obtained from uninfected explants grown on non-selective medium. Once transferred to soil, all T0-lines were genotyped and attentively observed for possible abnormalities of vegetative and reproductive organs, but no obvious consistent deviations were observed. Even though many fruits in all three groups were parthenocarpic, enough seed could be obtained from both transgenic and control lines to evaluate *TM8* function in the T1 generation, which was not possible using VIGS in *Nicotiana*. The two 35S:*TM8* and 14 selected *TM8*-RNAi transgenic T1-lines were genotyped and subsequently tested for altered *TM8* expression by RT-PCR (Additional file 7).

Based on the results in *Nicotiana* we expected that altered expression of *TM8* could result in a change of flowering time or number of leaves before floral initiation, however, this was not what we observed (Fig. 4c). Only one of the 14 tested *TM8*-RNAi lines was on average late flowering compared to control, and one 35S:*TM8* line was early flowering, opposite to what we observed in *Nicotiana*, but more important, not consistent with any of the other lines. Aside from flowering time, we attentively phenotyped the first three flowers of each T1 plant for deformed stamen cones, extra floral organs and other morphological abnormalities. Again, while the absolute majority of these flowers appeared normal, a few control, *TM8*-RNAi flowers and 35S:*TM8* flowers had a split stamen cone (Fig. 4c, d). Only three flowers, one in each group, showed this in a severe form as described by [9] (Fig. 4c, Additional file 8). Extra floral organs as seen in *Nicotiana* were also observed, but again in very low numbers and both in control and in *TM8*-RNAi and 35S:*TM8* lines (Fig. 4c, Additional file 8).

Together, we conclude that transgenic tomato plants overexpressing *TM8* or silencing *TM8* do not differ from controls and that no obvious function can be concluded for tomato. Our data suggest that *TM8* does not act as a flowering repressor in *Solanum lycopersium,* as it does in *Nicotiana*.

### Expression profiles throughout evolution are diverse

**Fig. 5:**
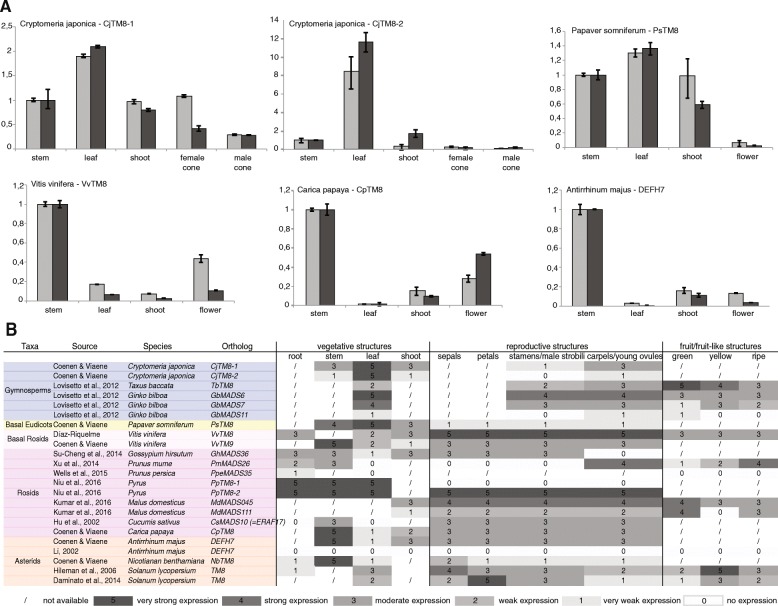
*TM8* expression profiles. **a** Relative expression of *TM8* in *Cryptomeria japonica*, *Papaver somniferum*, *Vitis vinifera*, *Carica papaya* and *Antirrhinum majus* in two biological replicates. Error bars represent standard deviations. **b** *TM8* expression throughout evolution comprising all expression data to date

Although we cannot generalize a repressor function for *TM8* to all Solanaceae, we asked if we could find a general pattern of expression throughout evolution. We compared available data from the literature to newly generated expression of *TM8* in *Cryptomeria japonica*, *Papaver somniferum*, *Vitis vinifera*, *Carica papaya* and *Antirrhinum majus* (Fig. 5). Based on these data, we might cautiously say that throughout evolution expression in leaves decreased, while expression in all other tissues varies too much or not enough data is available yet to draw any conclusions. In general, these data support the idea that *TM8* function is not widely conserved.

## Discussion

While most MADS-box gene clades are well characterized in at least one model organism, only limited and often inconsistent findings have been published about the *TM8*-clade. In an effort to contribute new knowledge about *TM8*, we reconstructed its phylogenetic history in angiosperms and characterized the gene in three Solanaceae species.

The *TM8*-lineage is one of the more ancient MADS-box lineages as it was already present in the last common ancestor of the Spermatophytes [4]. We found that unlike most other MADS-box transcription factors, its duplicates were not retained after major duplication events like the ε and γ whole genome duplication and triplication, preceding the origin of the angiosperms and core eudicots respectively [33, 34]. In Solanaceae, which also underwent an additional whole genome triplication, *TM8* is again mostly present as a single copy gene. The lack of duplicates in angiosperms is probably the main reason why *TM8* was lost several independent times [6]. However, the fact that so many angiosperm species could flourish without it, suggests that its function became generally less necessary, that it became specific for certain environments or that its function rapidly evolved in angiosperms.

We have shown that in *Nicotiana benthamiana* the *TM8* orthologue *NbTM8* functions as a repressor of *miR172*. *NbTM8* expression levels are positively regulated by *NbSVP* and their protein products interact. Both transcription factors have overlapping expression patterns in the shoot. Similar as for *NbTM8*, we identified *NbSVP* to function as a repressor of *miR172*. While we are able to propose a function in *Nicotiana benthamiana* this function appears not to be strongly conserved in other closely related Solanaceae species. The lack of a clear phenotype in the *Petunia TM8*-mutant and both the *TM8* overexpression and RNAi transgenic tomato lines, suggests that *TM8* has no important function in these species. We must therefore conclude that *TM8* function has strongly diverged or was even lost between these closely related species, even though their protein sequences appear to be conserved.

It appears that both *SVP* and *TM8* are part of a regulatory module that rapidly evolved in Solanaceae species and may have broadly contributed to phenotypic diversity between species. Indeed, also *SVP* function diverged within Solanaceae. In the closely related *Nicotiana tabacum NtSVP* was found to regulate pedicel elongation [35]. In tomato the *SVP* homologue is *JOINTLESS* where it functions in abscission zone development and inflorescence architecture [36, 37]. In *Physalis species MFP2* regulates the inflated calyx after pollination [31]. Outside of Solanaceae, the *Antirrhinum majus* homologue of *SVP* is *INCOMPOSITA*, which prevents prophyll development and controls floral meristem identity [38].

For tomato, our results do not fully correspond to those found in a similar study performed by Daminato et al. [9]. The authors described morphological malformations in the androecia in three out of 22 *TM8* overexpression lines as the only macroscopic difference compared to wild-type tomatoes [9]. We observed the same phenotype together with other disturbances of the floral whorl patterning, both in OE, RNAi and control plants. It must be noted here that most flowers of all our T0-lines looked perfectly normal, and only a few flowers of a few plants displayed this anomaly, often in the first flowers to open. In Daminato et al., it was not specified how many flowers in the transgenic lines displayed this phenotype. Where normal wild-type tomatoes were used as control in Daminato et al., we used tomatoes obtained from uninoculated callus grown in vitro and we hypothesize that the malformations observed in both studies could possibly be due to somaclonal variation [39]. In addition, only two wild-type tomatoes were described as a control, and since the low prevalence of the phenotype, at least in our case, it is not unlikely that the authors therefore missed the presence of split stamen cones in wild-type lines in their study. Alternatively, the fact that we only generated two overexpression lines, may in turn explain why we didn’t observe a consistent splayed out stamen phenotype.

Another explanation could be that Daminato et al. used the longer splice variant (NM_001247176.2) for their construct which indeed seems to occur regularly throughout Solanaceae as seen in our alignment (Additional file 6). We used the shorter variant as this was the clone we picked up from our cDNA. The short variant shows a deletion from bp 142 to bp 155 (located in the last part of the K-domain) and occurs regularly in Solanaceae. Thus far, nothing has been reported on the possible differences between both splice variants. It is not unlikely that the indel influences higher order complex formation, as is proposed for the K3-domain, and therefore the protein lost its functionality [40]. To evaluate the effect of the shorter and longer splice variant, both should be evaluated in the same experimental setup.

Finally, we noticed several intriguing similarities with another MADS-box gene, *FLOWERING LOCUS C* (*FLC*). *FLC* plays a key role in the vernalization response in *Arabidopsis* [41]. It represses flowering pathway integrators *FLOWERING LOCUS T (FT)* and *SUPPRESSOR OF OVEREXPRESSION OF CONSTANS1 (SOC1).* It is epigenetically downregulated by cold, allowing the plant only to flower after a prolonged period of cold [42, 43]. A first similarity to *TM8* is that both genes share the same location in the genome, as it was recently described that both are syntenic to *SEPALATA3* (*SEP3*) [44, 45]. The *FLC* gene, however, originated later than *TM8*, and it has been suggested that the *FLC*-like genes could be derived from a *TM8* homolog in an ancestral angiosperm [44, 45]. Secondly, it is also notable that both genes are the MADS-box clades that were lost most frequently throughout angiosperms [6]. Interestingly, in most cases where one was lost, the other is still present, meaning that most genomes contain at least one of them. Thirdly, there seems to be a functional link as well: both have been assigned to be repressors of developmental transitions and flowering and act through regulation of the microRNA pathway, at least in *Nicotiana* regarding *TM8* [46]. Furthermore, both share SVP as their interaction partner during these repressing activities [47]. Like *FLC*, the function of *SVP* in the delay of the phase transitions is linked to temperature [48, 49]. Considering that both *FLC* and *SVP* are temperature dependent genes, it seems an interesting option to evaluate the effect of temperature on *TM8* functionality in future research.

## Conclusions

We aimed to resolve one of the remaining open questions about MADS-box gene functions. We were able to generate functional data in three related Solanaceae species, but it remains difficult to generalize a function within angiosperms or even Solanaceae. While *TM8* seems to perform a function as repressor of *miR172* in *Nicotiana benthamiana*, its function seems to have diverged quickly between members of the same family. It has been proposed before that *TM8* is a rapidly evolving gene [4] and our data are in line with this view.

## Methods

All primers used throughout the research are listed in Additional file 9. All constructs were verified by sequencing before usage.

### Phylogenetic analyses

Previously identified *TM8* accessions from various species were used to blast (blastn) in all Viridiplantae genomes available to date provided by Phytozome 12.0 [20] and the Sol Genomics Network [50] (Additional file 1). All resulting coding sequences were aligned using MAFT and an initial phylogenetic tree was built using PhyML as implemented in Geneious with the GTR substitution model and default parameters [51, 52]. All sequences that fell within the strongly supported *TM8*-clade (SH-like branch support = 1) were selected for further analysis. *SOC1* genes were used as an outgroup to root the tree. The *TM8*-sequences detected in fully sequenced genomes were supplemented with hits resulting from blastn searches in the NCBI NT, EST and TSA databanks and the oneKP platform to find representatives in all angiosperm orders [21]. The final data matrix consisting of 177 sequences was again aligned by MAFT, manually optimized and the most likely tree was constructed using PhyML with SPR tree topology search and the GTR substitution model and evaluated by bootstrap analysis with 100 replicates [53]. Complete phylogeny including all accession numbers is provided in the Additional file 2.

### Plant material and growth conditions

We received transgenic *Nicotiana benthamiana* lines overexpressing Arabidopsis miR-172a-1 from Xuemei Chen (UC Riverside, Mlotshwa et al. [30]). For *Solanum lycopersicum* the Micro-Tom cultivar was used in this study. For *Petunia hybrida* wild-type W138 and a W138-line with the 7 bp footprint of a *dTph1* insertion in the *PhTM8* start codon were used. All plants were grown at constant temperature (25 °C) and long day conditions (16 h light, 8 h dark). *N. benthamiana* and *Petunia* seeds were first sown in Jiffy Pellets and grown in a conviron growth cabinet. Later, they were planted in pots and moved to a growth chamber.

### RNA isolation and cDNA preparation

#### RNA isolation and reverse transcription

All sampled plant material was immediately frozen in liquid nitrogen and stored at − 80 °C. Total RNA was isolated using TRIzol® following manufacturer’s instructions (Invitrogen, Carlsbad, USA) and Dnase treated with TURBO DNA-free (Ambion, Austin, USA). RNA was reverse transcribed to cDNA using the GoScript reverse transcription system (Promega, Madison, USA). RNA quality was determined using the spectrophotometer, successful reverse transcription was tested by amplification of the *ACTIN* gene by PCR.

#### RNA isolation for stem-loop qRT-PCR for microRNA quantification

RNA was again isolated using TRIzol® but for RNA precipitation we used twice the volume of isopropanol and precipitated for 30 min at − 80 °C. cDNA was prepared using the AMV Reverse transcription kit (Promega, Wisconsin, U.S.A.). Following [54], we designed a custom stem-loop primer to specifically reverse transcribe microRNA172 and microRNA156. Reactions were performed in total RNA pools in the presence of an oligo-dT primer to reverse transcribe poly-A-tailed mRNA’s using AMV reverse transcriptase (Promega, Madison, US).

### Characterization of *NbTM8* and *NbSVP* in *Nicotiana benthamiana*

#### Cloning of *NbTM8* and *NbSVP*, *SOC1*- and *AP1*-homologs

The full length sequence of *NbTM8* was cloned from cDNA derived from floral buds based on the sequence of *NtTM8* (EB449747). A partial sequence of *SVP* in *N. benthamiana* was present in the EST-database of Genbank (EH369950). We used 3′RACE to clone the 3′-sequence of *NbSVP*. A forward primer was combined with an oligodT to amplify the 3′region. For *APETALA1* (*AP1*), an alignment of homologs from *Nicotiana tabacum* (*NAP1–2*, AF009127) and *Nicotiana sylvestris* (*NsMADS2*, AF068726) was constructed. Forward and reverse primers were selected to amplify a 231-bp region of *NbAP1*. (*SOC1*) Based on *SUPRESSOR OF CONSTANS1*-homologs from *Nicotiana tabacum* (*tobmads1*, X76188) and Solanum lycopersicon (BG599624), a 339-bp fragment of *NbSOC1* was amplified following a similar strategy. New sequences were added to GenBank.

#### qRT-PCR and developmental series

To examine the expression of selected genes and microRNA’s (*NbTM8*, *NbSVP*, *NbSOC1*, *NbAP1*, *miR156* and *miR172*), qRT-PCR was used. Real-time PCR was performed on a StepOne Plus apparatus (Applied Biosystems, Forster City, US) using Fast SYBR Green Master Mix (Applied Biosystems, Forster City, US). Primers were designed using the Applied Biosystems Primer Express software. Quantification of mature microRNA levels was performed using primers complementary to the stem-loop primers in combination with microRNA forward complementary primers. To quantify genes or microRNA’s in EV, VIGS or overexpression lines, inflorescence tissue of plants before they started to flower was used. All data presented here are three technical replicates from two biological replicates and are normalized against *ACTIN* expression. The developmental series was started from seedlings with two fully expanded cotyledons. A subsequent stage was sampled every time a new leaf had emerged. To measure expression in separate plant organs, whole organs of mature plants were sampled at anthesis of the first flower. In the developmental series and separate plant organs, pooled plant material was collected to obtain enough tissue for RNA isolation. Data were analyzed using the delta CT-method.

#### In situ hybridization

Sense and Antisense probes were in vitro transcribed using T7 RNA polymerase (New England Biolabs, Ipswich, AU) in the presence of digoxigenin-labeled UTP (Roche, Basel, CH) from PCR amplified templates that included a T7 promoter. Tissues were fixed in 4% paraformaldehyde, paraffin embedded and sectioned at 8 μm. Sections were mounted on Probe-On-Plus slides (Fisher Scientific, Pittsburgh, US). Prehybridization, hybridization and detection were essentially following Carr & Irish [55].

#### Virus induced gene silencing of *NbTM8* and *NbSVP*

Gene-specific regions of *NbTM8* and *NbSVP* were introduced into the TRV2 vector [56]. The constructs were transformed into *Agrobacterium* strain GV3101 and used to infiltrate *N. benthamiana*. To quantify flowering time, *N. benthamiana* seeds were sown in long day conditions. A considerable number of plantlets (20–50) were infiltrated at the youngest possible stage (three to four leaf-stadium). After infiltration, plants were returned to their growth chamber and covered with foil for 2 days to keep them humid. Flowering time was counted as the number of leaves before the first flower appeared. As a control, plants were infiltrated with empty vector TRV2 and grown under the same conditions. Leaf and floral material from *NbTM8*-VIGS, *NbSVP*-VIGS plants showing phenotypes and empty vector control plants were collected in liquid nitrogen to check for effective downregulation.

#### Overexpression of *NbTM8* and *NbSVP*

Full length sequences of *NbTM8* and *NbSVP* from *Nicotiana benthamiana* were cloned into a 35S overexpression vector (pcB301, Filip Rolland). overexpression vectors were transformed into *Agrobacterium* strain GV3101. Stable transformation of *Nicotiana benthamiana* followed a transformation protocol optimized for tomato but using leaf-disks as explants [57]. Phenotypic characterization was performed in the T0 generation in comparison to T0 empty vector lines as strong 35S:*NbTM8* lines were fully female sterile.

#### Yeast two-hybrid

Full length *NbTM8* and *NbSVP* sequences were fused with the GAL4 activation domain in the pGAD424-vector and the GAL4 DNA-binding domain in the pGBT9 vector (Clontech, Mountain View, CA). Each possible vector combination was transformed in yeast strain Y187 as described in [58]. Two colonies per transformation were used for β-galactosidase liquid assays, and interaction was detected by use of ortho-Nitrophenyl-β-galactoside (ONPG) as a substrate [59]. Miller units as a quantification of β-galactosidase activity was calculated using the following formula: Miller units = (1000 × A420) / (t × V × OD600) with A420 = absorbance at 420 nm, OD600 = Optical density at 600 nm, t = number of minutes and V = 0. 5 mL.

#### Co-IP

Full length *NbTM8* and *NbSVP* were cloned in the HBT95 expression vector [60] in frame with a double hemagglutinin (HA) or FLAG tag and subsequently maxi-prepped. Afterwards they were transformed in *Arabidopsis* protoplasts using PEG-Ca^2^ transformation and co-immunoprecipitated as described in [61]. Proteins were captured by agarose beads with a FLAG-antibody and co-immunoprecipitated proteins were visualized through Western Blot using HA-HRP and FLAG-HRP antibodies.

#### Scanning electron microscopy

The plant material was fixed in FAA (70% ethanol:acetic acid:40% formaldehyde, 90: 5: 5) and washed twice in 70% ethanol and dehydrated in a 1: 1 mixture of 70% ethanol and dimethoxymethan (DMM) for 5 min and in pure DMM for 20 min. After critical-point drying (CPD 030;BAL-TEC AG, Balzers, Liechtenstein), the dried material was mounted on aluminum stubs using Leit-C and coated with gold (SPI Module Sputter Coater; Spi Supplies, West Chester, PA, USA) before observation with a JEOL JSM-6360 SEM (Jeol Ltd., Tokyo, Japan).

### *PhTM8* characterization in *Petunia hybrida*

#### Identification of the *phtm8* dTph1 insertion allele and derived footprint allele

The *phtm8 dTph1* insertion allele was identified by BLAST-searching a *dTph1* transposon flanking sequence database [62], that has been considerably expanded in recent years. The presence of the *dTph1* insertion was confirmed in planta in off-spring of the in silico identified insertion line, by PCR using a gene-specific primer pair flanking the insertion site. Among the different progenies, we identified several plants that were homozygous mutant for a putative footprint allele. Sequencing of the footprint allele showed a 7 bp footprint insertion causing a frameshift mutation in the *PhTM8* coding sequence. Progeny from homozygous WT and homozygous footprint mutants were obtained by selfing, and used for further phenotypic analysis.

#### Phenotyping of the *Petunia phtm8* footprint mutant

Twenty-one W138 *Petunia* wild-types (WT) and 19 *phtm8* footprint mutants were numbered and placed randomly together in the growth chambers. Plants were subsequently phenotyped for general anomalies and vegetative characteristics. ‘Days to flowering’ was counted from the day they were sown, till the day of full anthesis of the first flower. ‘Number of leaves’ include all true leaves (no cotyledons) before the first inflorescence. Upon flowering, flower characteristics like number of sepals, number of petals together with other anomalies of the first 20 flowers of each plant or until the end of the experiment were counted. Non-paired two-tailed t-tests were applied to determine if the means of the measured characteristics were significantly different between the control group and the footprint lines.

#### Cloning of *PhTM8*

Two *PhTM8* primer-pairs were designed based on the predicted CDS of *PhTM8* (received from M. VandenBussche). In addition *PhTM8* forward primers were combined with polyT reverse primers. PCR was carried out on cDNA from whole plant material of 6, 8, 12, 14, 16, 18 and 20 leaves stadia, in inflorescence stadium and flowering stadium with annealing temperatures ranging between 47 °C and 60° for 30–45 cycles. All resulting amplicons of the proper length (around 579 bp) were cloned into the pGEM®-T Easy (Promega) and subsequently sequenced, but non matched with *TM8*. After our failed attempts to clone *PhTM8*, following datasets were blasted for *PhTM8* expression: *Petunia* transcriptome [63], Unigenes and ESTs at the SOL Genomics Network (SGN) [50] and NCBI Transcriptome Shotgun Assembly (TSA) for Petunia species.

### Characterization of *TM8* in *Solanum lycopersicum*

#### Cloning of *TM8*

Full-length tomato *TM8* was cloned from mature leaf cDNA using primers based on the sequence (X60760.1) [7]. After confirmation by sequencing, it was cloned into pDONR21 by BP clonase reaction and subsequently cloned into the pK2GW7 vector by LR clonase reaction from Gateway Cloning [64]. For the RNAi-construct a 332 bp long part of tomato *TM8*, starting from bp 266 in the I-domain and ending in the 3′UTR, was selected. The short *TM8*-fragment was blasted in the tomato genome to verify its specificity. No hits other than *TM8* were found, proving that the selected sequence is highly specific and suited for RNAi. The 332 bp sequence was cloned into the pK7GWIWG2(I) vector. All destination vectors were confirmed by sequencing. The constructs were transformed into the LBA4404 *Agrobacterium* strain and selected on spec/strep/rif plates.

#### Tomato transformations

The tomato transformation protocol was followed as in [65]. Tomato cotyledons were cut in half and used as explants for Agro-infection. Transformed explants were further grown on 2Z-media supplemented with kanamycin (kan), allowing only successfully transformed explants to survive. Control lines were generated from non-transformed callus and grown on 2Z-medium without antibiotics. After shoot and root generation on MSSV +IBA + kan medium, transformed plants were planted in soil. All T0 and T1 plants were genotyped to confirm the presence of the constructs using a forward primer in the 35S promotor and a reverse primer in the K-domain (Primer List). Semi-quantitative PCR confirmed much higher expression in OE lines compared to ctl, and no (or much lower) expression could be detected in silenced lines. Inflorescence with one open flower were sampled for the RT-PCR.

#### Phenotyping of tomato transformants

All T0 plants were observed for abnormalities in vegetative or reproductive structures. Seed was harvested where possible and sown out again. T1 plants were subjected to elaborate phenotyping. Forty control, 40 RNAi and 36 OE individuals were numbered and randomly located in the growth chamber. Plants were phenotyped in the same way as *Petunia*. The first three flowers of each plant were observed for number of petals, sepals, split stamen cones or other abnormalities. Statistical significance was determined by an unpaired two-tailed t-test.

## Additional files

Additional file 1: List of blasted genomes. (XLSX 42 kb)

Additional file 2: *TM8* alignment and complete phylogeny with accession numbers. (NEWICK 19 kb)

Additional file 3: In situ hybridization sense probe negative control. Sam: shoot apical meristem; f: flower meristem; df; developing flower. (PDF 3584 kb)

Additional file 4: Characterization of developmental phases in *Nicotiana benthamiana.* A) Juvenile-adult phase change in *Nicotiana* occurs around leaf 3–4 as indicated by the appearance of large trichomes on the leaf disk (indicated by black dots) and a pointed leaf tip. B) Detail of a large trichome indicative for the adult phase in *Nicotiana*. C) Trichome density increases faster in two *Nicotiana* transgenic lines overexpressing *miR172* (L6 and L7). D) Relative expression of *NbAP1* (left) and *NbSOC1* (right) during *Nicotiana* development (cotyledons until the 14th leaf). The corresponding gray and black arrows indicate respectively floral transition and initiation of floral development. (PDF 11213 kb)

Additional file 5: Illustration of footprint mutant sequence. Wild-type *PhTM8* sequence around the start codon compared to the *phtm8* sequence around the new startcodon created by a *dTph1* transposon insertion. The *dTph1* transposable element left a 7 bp footprint (underlined) creating a new start codon (red) which leads to an immediate frameshift *PhTM8*. The newly translated protein of the footprint mutant results quickly in a stop codon leading to a short non-sense protein. (PNG 21 kb)

Additional file 6: *TM8* protein alignment of Solanaceae reveals different splice variants. *PhTM8* aligns well to its orthologs and does not show any sign of pseudogenization. (PDF 1315 kb)

Additional file 7: RT-PCR of *TM8* and *ACTIN* in tomato in T1-phenotyped lines. Control, *TM8* overexpression (OE) and *TM8*-RNAi lines (31 cycles all starting from 10 ng/μL cDNA obtained from inflorescences with at least one open flower). (PDF 46 kb)

Additional file 8: Abnormalities observed in callus cultivated tomato plants. A) normal wild-type flower consisting of five petals, five sepals, a cone formed by five stamens and an ovary within. B) Control flower with interrupted and sepaloïd stamen cone. C) RNAi flower with extra floral organs and crown anthers. D) OE flower with sepaloïd petals, E) split stamen cone, F) fusion of stamen and pistil, and G) disturbed growth and leaf morphology. H) RNAi flower with splayed out stamen cone I) OE flower with sepaloïd stamen. In picture E) and F) petals and sepals were removed for better sight on stamens and pistil. (PDF 202 kb)

Additional file 9: Primer list. (XLSX 43 kb)

## Abbreviations

AP1: APETALA1
AP2: APETALA2
FLC: FLOWERING LOCUS C
FT: FLOWERING LOCUS T
OE: Overexpression
SOC1: Suppressor of constans 1
SVP: Short vegetative phase
TM8: Tomato MADS 8
VIGS: Virus induced gene silencing
