# Supplementary figures and images for: TM8 represses developmental timing in Nicotiana benthamiana and has functionally diversified in angiosperms
Source: BMC Plant Biol. 2018 Jun 22;18:129. doi: 10.1186/s12870-018-1349-7 (PMC6013966; doi:10.1186/s12870-018-1349-7)

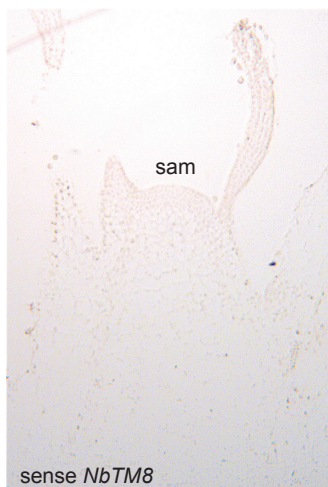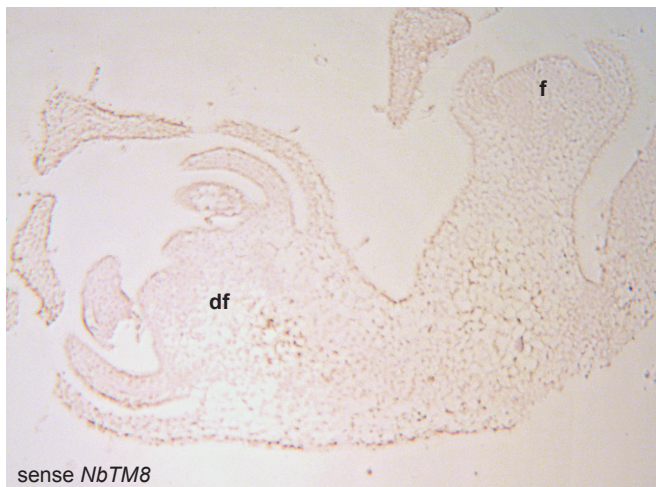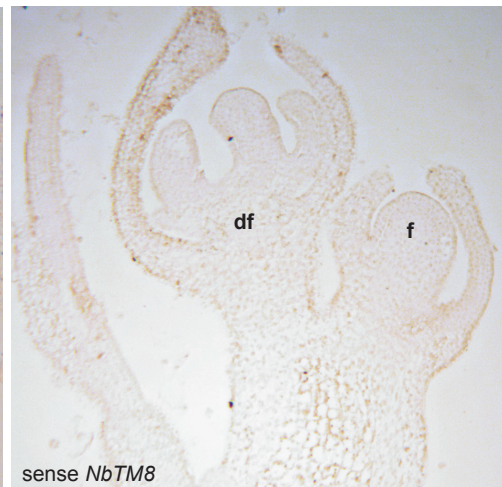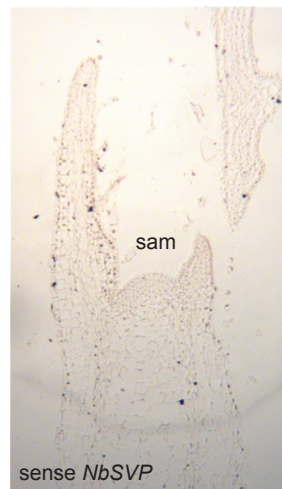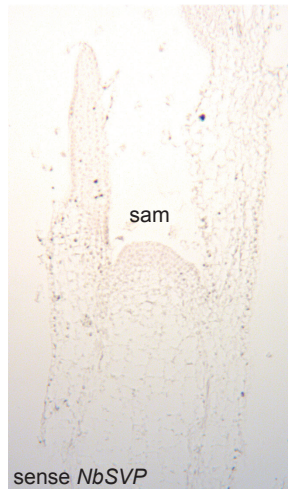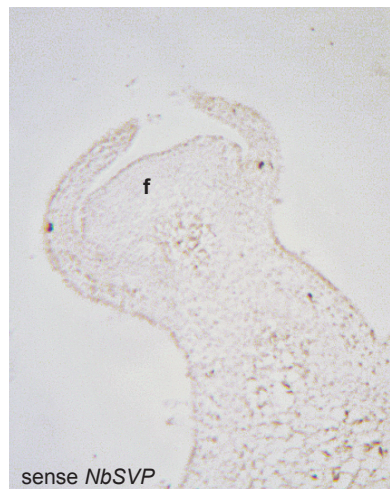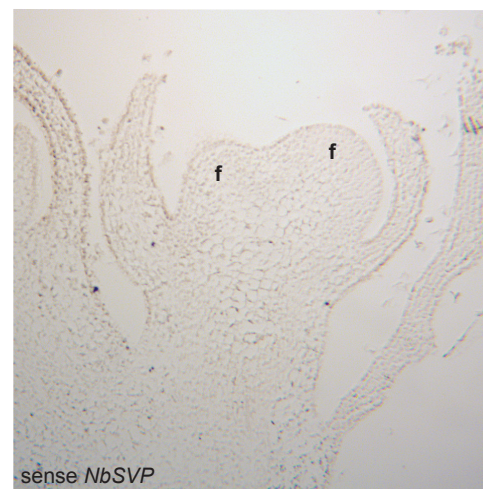

Supplement: Supplementary file 3 — In situ hybridization sense probe negative control. Sam: shoot apical meristem; f: flower meristem; df; developing flower. (PDF 3584 kb) [file 12870_2018_1349_MOESM3_ESM.pdf]

**A**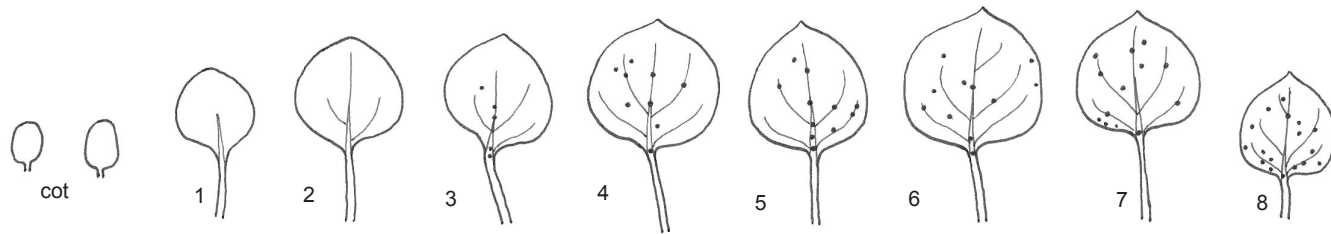**B**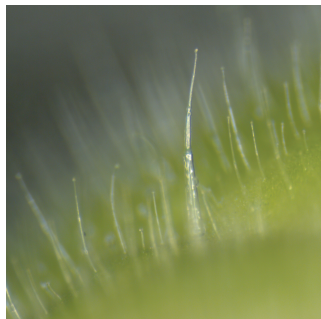**C**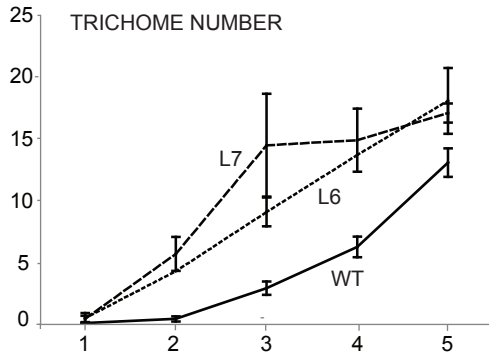**D**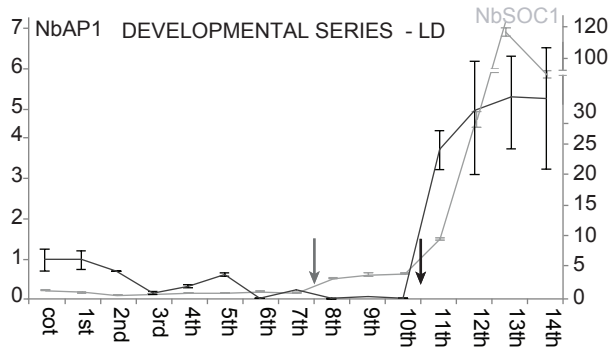

Supplement: Supplementary file 4 — Characterization of developmental phases in Nicotiana benthamiana. A) Juvenile-adult phase change in Nicotiana occurs around leaf 3–4 as indicated by the appearance of large trichomes on the leaf disk (indicated by black dots) and a pointed leaf tip. B) Detail of a large trichome indicative for the adult phase in Nicotiana. C) Trichome density increases faster in two Nicotiana transgenic lines overexpressing miR172 (L6 and L7). D) Relative expression of NbAP1 (left) and NbSOC1 (right) during Nicotiana development (cotyledons until the 14th leaf). The corresponding gray and black arrows indicate respectively floral transition and initiation of floral development. (PDF 11213 kb) [file 12870_2018_1349_MOESM4_ESM.pdf]

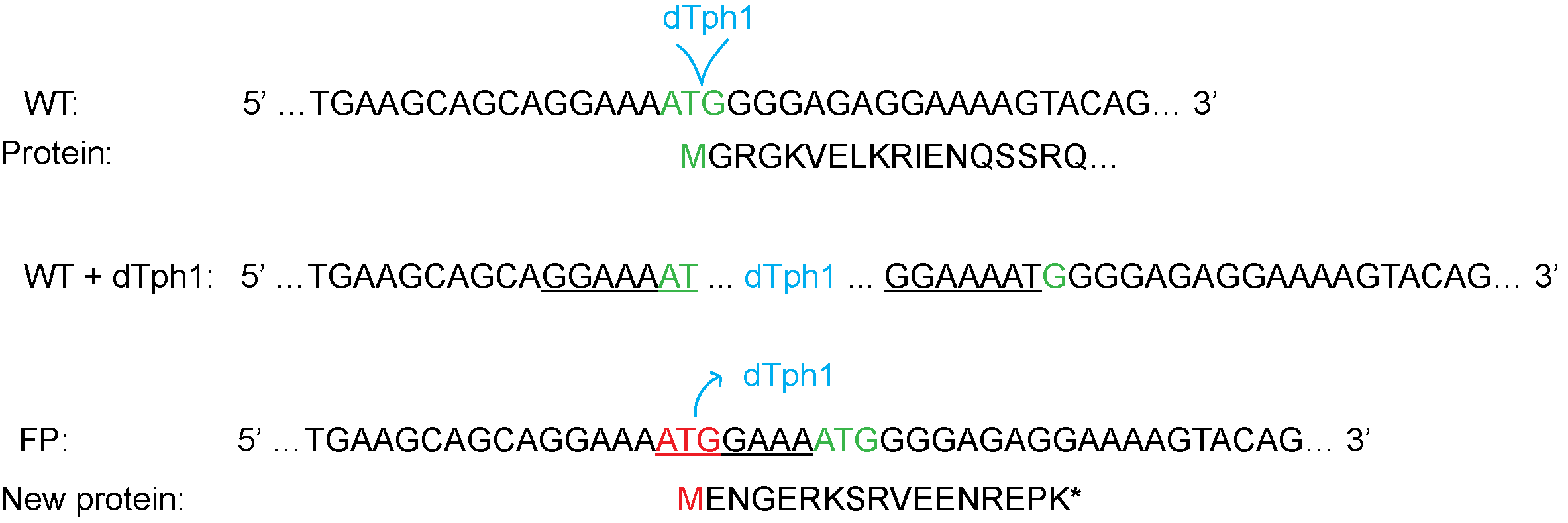

Supplement: Supplementary file 5 — Illustration of footprint mutant sequence. Wild-type PhTM8 sequence around the start codon compared to the phtm8 sequence around the new startcodon created by a dTph1 transposon insertion. The dTph1 transposable element left a 7 bp footprint (underlined) creating a new start codon (red) which leads to an immediate frameshift PhTM8. The newly translated protein of the footprint mutant results quickly in a stop codon leading to a short non-sense protein. (PNG 21 kb) [file 12870_2018_1349_MOESM5_ESM.png]

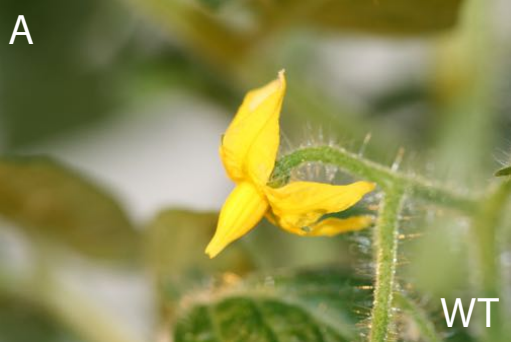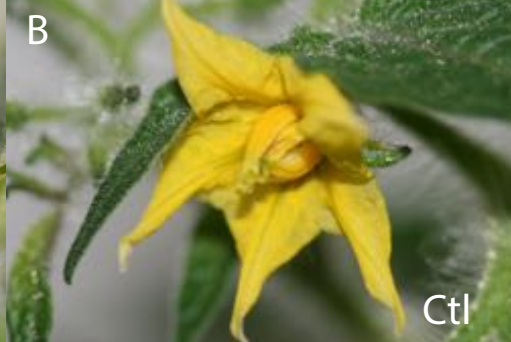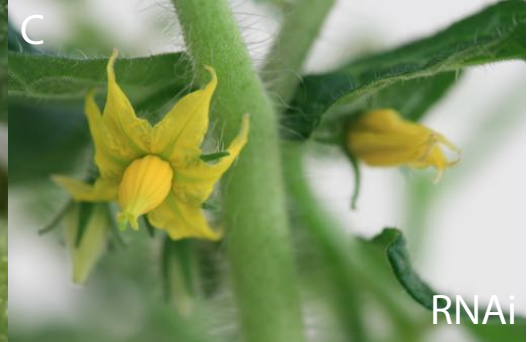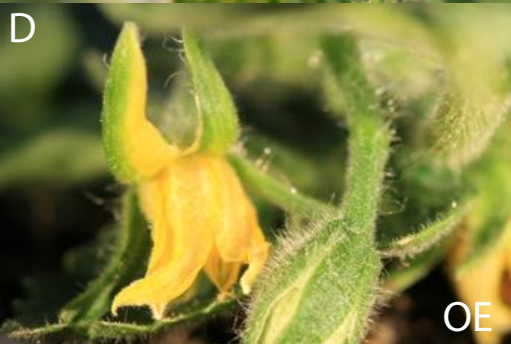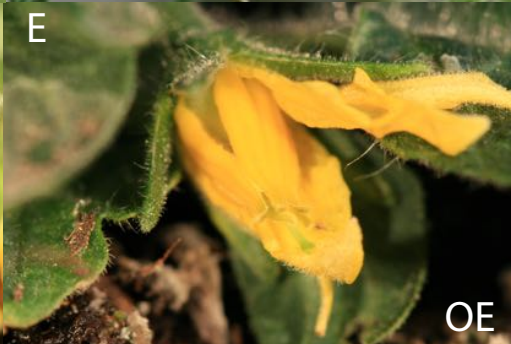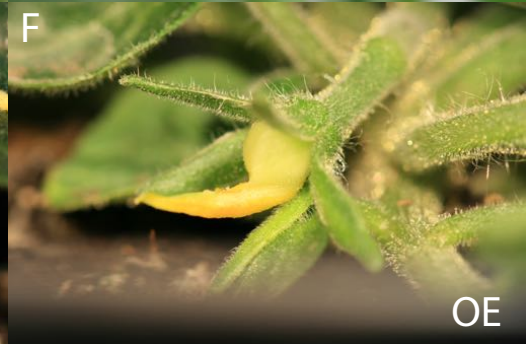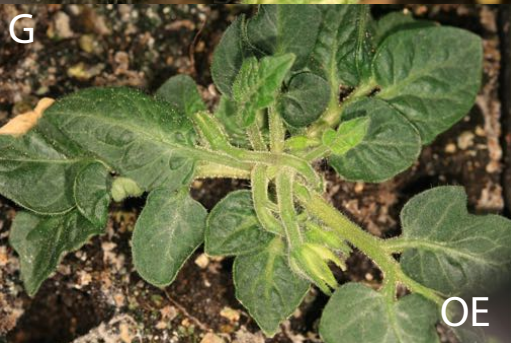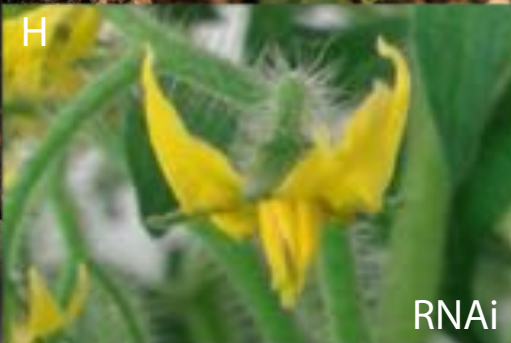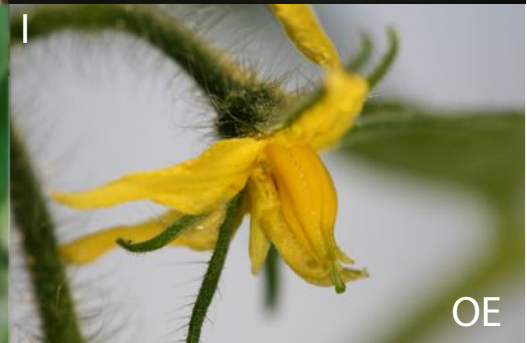

Supplement: Supplementary file 8 — Abnormalities observed in callus cultivated tomato plants. A) normal wild-type flower consisting of five petals, five sepals, a cone formed by five stamens and an ovary within. B) Control flower with interrupted and sepaloïd stamen cone. C) RNAi flower with extra floral organs and crown anthers. D) OE flower with sepaloïd petals, E) split stamen cone, F) fusion of stamen and pistil, and G) disturbed growth and leaf morphology. H) RNAi flower with splayed out stamen cone I) OE flower with sepaloïd stamen. In picture E) and F) petals and sepals were removed for better sight on stamens and pistil. (PDF 202 kb) [file 12870_2018_1349_MOESM8_ESM.pdf]
