# Supplementary material for: TM8 represses developmental timing in Nicotiana benthamiana and has functionally diversified in angiosperms
Source: BMC Plant Biol. 2018 Jun 22;18:129. doi: 10.1186/s12870-018-1349-7 (PMC6013966; doi:10.1186/s12870-018-1349-7)
Supplement: Supplementary file 7 — RT-PCR of TM8 and ACTIN in tomato in T1-phenotyped lines. Control, TM8 overexpression (OE) and TM8-RNAi lines (31 cycles all starting from 10 ng/μL cDNA obtained from inflorescences with at least one open flower). (PDF 46 kb) [file 12870_2018_1349_MOESM7_ESM.pdf]

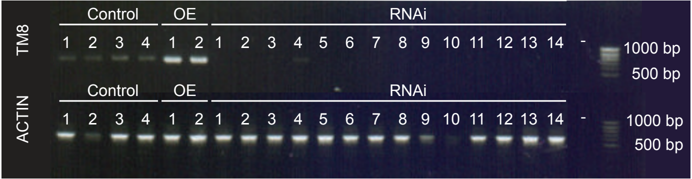
TM8
Control
OE
RNAi
1 2 3 4 1 2 1 2 3 4 5 6 7 8 9 10 11 12 13 14 -
1000 bp
500 bp
ACTIN
Control
OE
RNAi
1 2 3 4 1 2 1 2 3 4 5 6 7 8 9 10 11 12 13 14 -
1000 bp
500 bp
